# Novel mechanisms for organic acid-mediated aluminium tolerance in roots and leaves of two contrasting soybean genotypes

**DOI:** 10.1093/aobpla/plx064

**Published:** 2017-11-17

**Authors:** Shou-Cheng Huang, Shu-Juan Chu, Yu-Min Guo, Ya-Jing Ji, Dong-Qing Hu, Jing Cheng, Gui-Hua Lu, Rong-Wu Yang, Cheng-Yi Tang, Jin-Liang Qi, Yong-Hua Yang

**Affiliations:** Institute of Plant Molecular Biology, State Key Laboratory of Pharmaceutical Biotechnology, School of Life Sciences, Nanjing University, Nanjing, China; Jiangsu Collaborative Innovation Center for Modern Crop, Nanjing Agricultural University, Nanjing, China; College of Life Science, Anhui Science and Technology University, Fengyang, China

**Keywords:** Aluminium tolerance, citrate metabolism, jasmonic acid, soybean, transcriptome

## Abstract

Aluminium (Al) toxicity is one of the most important limiting factors for crop yield in acidic soils. However, the mechanisms that confer Al tolerance still remain largely unknown. To understand the molecular mechanism that confers different tolerance to Al, we performed global transcriptome analysis to the roots and leaves of two contrasting soybean genotypes, BX10 (Al-tolerant) and BD2 (Al-sensitive) under 0 and 50 μM Al^3+^ treatments, respectively. Gene Ontology and Kyoto Encyclopedia of Genes and Genomes analyses revealed that the expression levels of the genes involved in lipid/carbohydrate metabolism and jasmonic acid (JA)-mediated signalling pathway were highly induced in the roots and leaves of both soybean genotypes. The gene encoding enzymes, including pyruvate kinase, phosphoenolpyruvate carboxylase, ATP-citrate lyase and glutamate-oxaloacetate transaminase 2, associated with organic acid metabolism were differentially expressed in the BX10 roots. In addition, the genes involved in citrate transport were differentially expressed. Among these genes, *FRD3b* was down-regulated only in BD2, whereas the other two multidrug and toxic compound extrusion genes were up-regulated in both soybean genotypes. These findings confirmed that BX10 roots secreted more citrate than BD2 to withstand Al stress. The gene encoding enzymes or regulators, such as lipoxygenase, 12-oxophytodienoate reductase, acyl-CoA oxidase and jasmonate ZIM-domain proteins, involved in JA biosynthesis and signalling were preferentially induced in BD2 leaves. This finding suggests that the JA defence response was activated, possibly weakening the growth of aerial parts because of excessive resource consumption and ATP biosynthesis deficiency. Our results suggest that the Al sensitivity in some soybean varieties could be attributed to the low level of citrate metabolism and exudation in the roots and the high level of JA-mediated defence response in the leaves.

## Teaser

Aluminium (Al) toxicity is one of the most important limiting factors for crop yield in acidic soils. However, the mechanisms that confer Al tolerance remain largely unknown. Based on the global transcriptome analysis of the roots and leaves of two contrasting soybean genotypes, BX10 (Al-tolerant) and BD2 (Al-sensitive) under 0 µM and 50 µM Al3+ treatments, our findings suggest that BX10 can resist Al by secreting additional citrate into the rhizosphere from the roots to chelate Al and by avoiding a JA-mediated defense response that allows resource allocation to maintain leaf growth.

## Introduction

Aluminium (Al) is one of the major restricting factors for crop production in acidic soils (pH < 5.0) (Foy 1988). Over 50 % of global arable land is identified as acidic soils (Bot *et al.* 2000). In China, acidic soils constitute about 20 % of the total land area. Al toxicity leads to the inhibition of root growth and subsequently the poor uptake of water and minerals (Kochian 1995).

Different plant species or different genotypes within the same species have evolved special mechanisms to alleviate Al toxicity and survive in high Al environments. Exudation of organic acids, such as citrate, malate and oxalate from roots, is one of the most important mechanisms to chelate Al in rhizosphere (Ma 2000). Some related genes, including aluminium-activated malate transporter (*ALMT1*) (Delhaize *et al.* 2004; Sasaki *et al.* 2004) and multidrug and toxic compound extrusion (*MATE*) family members (Magalhaes *et al.* 2007), are responsible for excretion of malate and citrate, respectively. *GmALMT1* encodes a malate transporter in soybean and is required for the adaptation to Al toxicity by regulating malate efflux (Liang *et al.* 2013).

In soybean, the Al-triggered organic acid secretion from roots is required for Al detoxification; differential exudation is observed in different soybean genotypes, that is, the tolerant cultivars generally secrete more organic acids than the sensitive cultivars (Silva *et al.* 2001; Dong *et al.* 2004; Liao *et al.* 2006). However, aerial proportions are inevitably involved in Al stress response because of the continuous translocation and accumulation of Al in the above-ground structures of plant species. Transcriptome analysis showed that rice leaves exhibit specific responses to abiotic stresses (Minh-Thu *et al.* 2013; Zhou *et al.* 2016).

Soybean is an important oil-bearing crop worldwide and is largely cultivated in acidic soils. As the first country to domesticate soybeans, China is rich in soybean germplasm resources with large variations of Al tolerance (Bo *et al.* 2007). However, Al toxicity restrains the growth of soybeans by inhibiting root elongation, reducing root activity and reducing leaf photosynthesis (Liu *et al.* 2004; Zhang *et al.* 2007). The mechanisms underlying different Al responses in soybean genotypes with contrasting Al tolerance are not yet fully understood at the molecular level. Furthermore, no studies have investigated the transcriptional response to Al stress in the leaves of soybeans with different Al tolerance. BX10 soybean, which originated from Brazil, is more resistant to Al than BD2 soybean, which originated from Guangdong, China (Xu 2003; Dong *et al.* 2004). These two soybean genotypes have been intensively studied because of their different Al-tolerance properties (Dong *et al.* 2004; Li *et al.* 2012; Yang *et al.* 2012). The former exhibits lower root growth inhibition than the latter under Al treatment (Dong *et al.* 2004; Zhen *et al.* 2009). Although BX10 secretes more citrate than BD2 after exposure to Al stress (Dong *et al.* 2004; Binbo *et al.* 2009), the molecular characterizations for organic acid metabolism and transport of these two soybeans should be clarified with further evidence. In this work, a genome-wide transcriptional analysis was performed to discover the differences in organic acid metabolism by coordinately using the roots and leaves as materials. This study aimed to elucidate the possible mechanisms conferring different Al tolerance in two soybean genotypes.

## Methods

### Plant growth and treatments

Two soybean genotypes (*Glycine max*), namely, an Al-tolerant cultivar BaXi10 (BX10) that originates from Brazil and an Al-sensitive cultivar BenDi2 (BD2) that comes from Guangdong Province of China, were employed in this study. The plump seeds were subjected to surface sterilization in 0.1 % mercuric chloride for 10 min and then rinsed five times with distilled water. The seeds were soaked in sterilized distilled water overnight and kept at 25 °C in the dark for germination. After 4 days, the seedlings were transplanted into 1/2 Hoagland nutrient solution in the chamber for 1 week under the following conditions: 26 °C/22 °C (day/night) for 16 h photoperiod (a photon flux density of 400 mmol m^−2^ s^−1^), 70 % relative humidity. The uniformly grown seedlings were then transferred into proper vessels containing 100 μM CaCl_2_ (pH 5.0) with either 0 (−Al) or 50 μM AlCl_3_ (+Al) solutions. Eight samples (four root samples and four leaf samples) were harvested at 48 h, and 0–1 cm root tips were collected, frozen in liquid nitrogen and kept at −80 °C for RNA extraction. The samples were pooled by genotype and treatment [e.g. BX10+Al (root), BX10−Al (root), BD2+Al (root), BD2−Al (root), BX10+Al (leaf), BX10−Al (leaf), BD2+Al (leaf) and BD2−Al (leaf)] to minimize the inter-individual differences.

### Root length measurement

Root length was measured by inserting the root into a transparent plastic tube and observing its appearance until 72 h. Relative root length (RRL) is the mean of 100 × (net growth in a treatment solution)/(net growth in the control) (Ryan *et al.* 1995). Two-tailed *t*-test was performed to determine the significance between the RRL of two genotypes at the same treatment time.

### RNA isolation, library construction and high-throughput sequencing

Total RNA was isolated by using Trizol reagent (Takara, Dalian, China). The mRNAs were purified by using Dynabeads Oligo(dT)_25_ mRNA isolation beads (Thermo Fisher Scientific Inc., USA). mRNA fragmentation was conducted by physical and chemical approaches, and the mRNA fragments of about 155 bp in length were collected. The obtained mRNAs were immediately reverse transcribed into first-strand cDNA and then used for second-strand cDNA synthesis. After the end reparation and 3′ A-tailing processes, the double-strand cDNAs were ligated to special adaptors. The products were purified using AMPureXP beads (NEB, USA), and each library was normalized by adjusting the cDNA concentration to 10 nM before subjecting to high-throughput sequencing on an Illumina HiSeq 2000 sequencer at Personalbio Co., Ltd (Shanghai, China).

### Analysis of high-throughput sequencing data

The raw reads were filtered using FastQC package to discard contaminant sequences and low quality reads (phred quality score < 30 and read length < 50 bp). The obtained clean reads were mapped to reference genome (Glyma1.0) using Bowtie/Tophat program (http://tophat.cbcb.umd.edu/). BLASTp searches (*E*-value < 1e-5) were conducted for the following databases: Ensembl, JGI, Kyoto Encyclopedia of Genes and Genomes (KEGG) and eggNOG to find the corresponding transcripts in soybean genome and acquire the annotations of these transcripts. Expression abundance was normalized by using reads per kilo bases per million reads (RPKM).

### Differential expression analysis, and Gene Ontology and KEGG enrichment analysis

DEGSeq was utilized to identify the differentially expressed genes (DEGs) in each pairwise comparison (Wang *et al.* 2010). False discovery rate (FDR) < 0.05, and log2 ratio of each gene expression > 1 or < −1 (+Al/−Al) were used as the threshold to identify the significance of the difference for each gene expression. Functional annotation was performed against Gene Ontology (GO) (http://geneontology.org/) database. KEGG pathway enrichment analysis was conducted against the KEGG database by using the differentially expressed transcripts with KEGG ORTHOLOGY (KO) accession numbers.

### Real-time PCR validation

In brief, 1.0 μg of total RNA was reversely transcribed using GoScript™ Reverse Transcription System (Promega, USA). The first-stand cDNA was used as template for real-time PCR with SYBR Fast qPCR Mix (Takara, Dalian, China). *GAPDH* mRNA was used as an internal control to normalize each gene expression. PCR was conducted on ABI 7500 Real-Time PCR System. Data were acquired with SDS software v2.0 (Applied Biosystems, USA). The 2^−ΔΔCt^ method was used to calculate the relative expression of the each gene (Livak and Schmittgen 2001). Two-tailed *t*-test was performed to compare the differences in the expression of paired samples. Three biological replicates were used, and the means were considered significantly different when *P* < 0.05.

## Results

### Morphological responses of two soybean genotypes under Al stress

**Figure 1.**
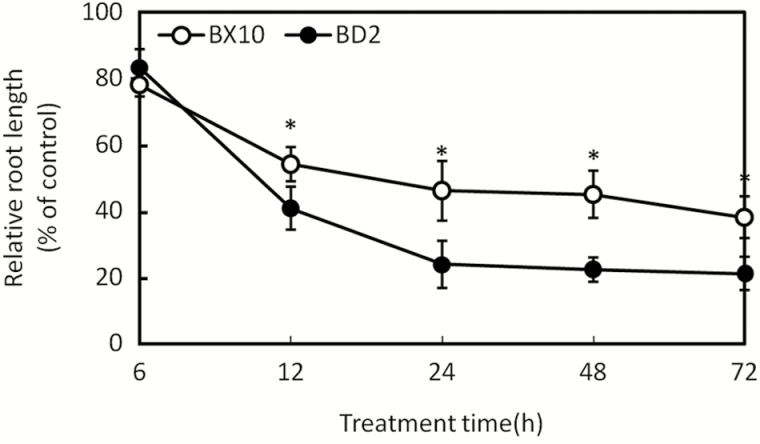
Time course of RRL of BX10 and BD2 soybeans under 0 or 50 μM Al^3+^ treatments. Error bars denote mean ± SD (*n* = 15). Asterisk (*) indicates significant difference at *P* < 0.05.

Root elongation inhibition is a typical response to Al toxicity in plants (Foy 1988). We determined RRL of two soybean genotypes exposed to Al^3+^ and contained solution at different time intervals. Under stress Al treatments, the growth of BD2 roots was greatly inhibited at different time periods after Al stress **[see Supporting Information—Fig. S1]**. The two soybean genotypes have significantly different root inhibition rates at 48 h of Al treatment (Fig. 1). These results clearly indicate that BX10 exhibits higher Al tolerance than BD2 in terms of root elongation.

### Overview of high-throughput sequencing results

Eight libraries from the roots and leaves of BX10 and BD2 soybean genotypes after 48 h treatment were constructed for high-throughput sequencing. Each library contained more than 54 million high-quality 100-bp pair-ended reads. Over 93 % of trimmed reads corresponded to the soybean reference genome (Schmutz *et al.* 2010). Among which, > 97 % was uniquely mapped, and > 91 % was mapped to genes. These findings suggest the fine quality of the RNA-seq results. Genome annotation was initiated by blasting against a series of open database, which allowed the annotation of 54175 transcripts (Table 1).

**Table 1.** Summary of RNA-seq reads mapped to soybean genome.

	Root				Leaf			
	BX10−Al	BX10+Al	BD2−Al	BD2+Al	BX10−Al	BX10+Al	BD2−Al	BD2+Al
Raw reads	76370154	88995142	86099746	64260978	54985492	70889914	108647888	119421598
Trimed reads	50689428	59117704	57933170	42823052	36814596	47292610	86951872	95824802
Mapped reads	47890719	55927457	53885648	40353198	35404671	45469878	83790325	92040829
Unique mapped	46820673	54492541	52643212	39457219	34286520	44131138	81489112	89494217
Multiple mapped	1070046	1434916	1242436	895979	1118151	1338740	2301213	2546612
Mapped to gene	46303509	53896105	51959290	38971445	33951147	43699034	80767625	88715040

### General effects of Al stress on gene expression in the roots and leaves of soybeans

**Figure 2.**
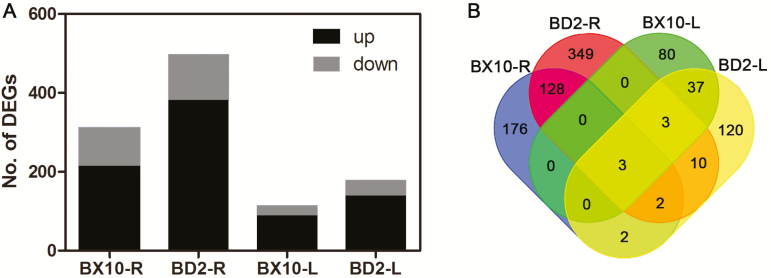
Screening of DEGs in the roots and leaves of two soybean genotypes. Up-/down-regulated genes (A) and multiple intercomparison (B) were shown. BX10-R and BX10-L denote DEGs in the roots and leaves of BX10, whereas BD2-R and BD2-L represent DEGs in the roots and leaves of BD2, respectively.

A total of 311 and 495 DEGs were screened in roots, whereas 122 and 176 DEGs were screened in the leaves of BX10 and BD2, respectively **[see Supporting Information—Table S1]**. Furthermore, the majority of DEGs was up-regulated under 48 h Al stress condition (Fig. 2A). A total of 133 transcripts showed common differential expression in the roots of BX10 and BD2, whereas 43 transcripts showed common differential expression in the leaves of BX10 and BD2 (Fig. 2B). Three transcripts revealed the Al response in the roots and leaves of these two soybean genotypes. Among these transcripts, two *ALS3* homologs were up-regulated.

**Figure 3.**
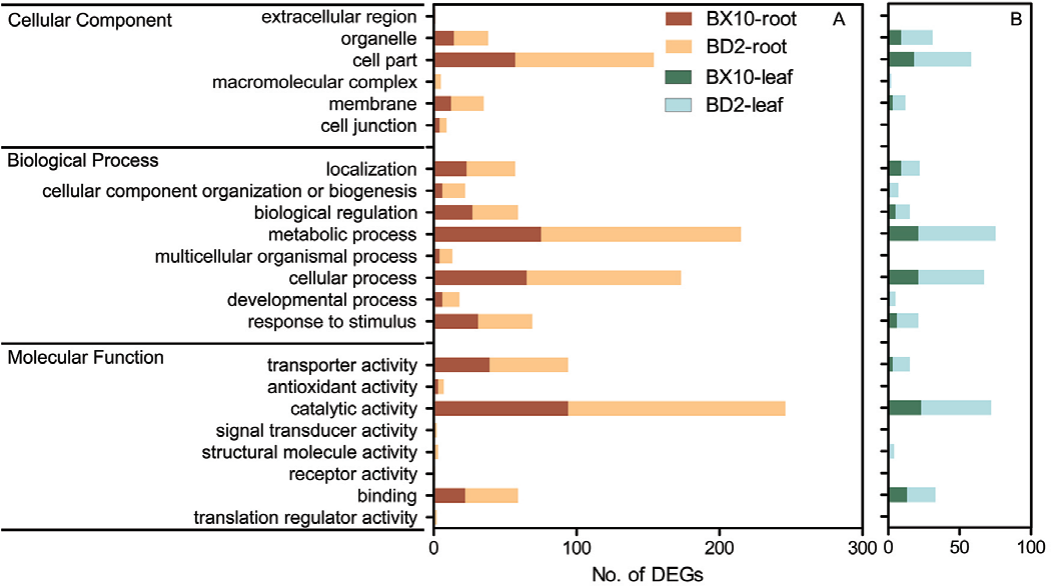
GO classification of DEGs in the roots (A) and leaves (B) of two soybean genotypes. ‘Cellular component’, ‘Biological process’ and ‘Molecular function’ categories were shown. Some categories are explained as follows: Binding, interacts selectively and non-covalently with substances, such as DNA, ATP, protein, etc.; Localization, positions a substance or cellular entity and maintains these in those locations; Cell part, cell component; Membrane, plasma or organelle membrane.

Gene Ontology enrichment analysis was used to explore the functional classifications of DEGs in the roots and leaves of these two soybean genotypes (Fig. 3). In the ‘Molecular function’ category, the genes involved in ‘Catalytic activity’, ‘Transporter activity’ and ‘Binding activity’ were over-represented. In the ‘Biological process’ category, the most represented group were ‘Metabolic process’, ‘Localization’ and ‘Cellular process’. However, the number of DEGs involved in metabolic process in the roots was larger than those in the leaves, confirming that the root is the major target of Al toxicity (Delhaize and Ryan 1995). With regard to the ‘Cellular component’ category, the ‘Cell part’, ‘Organelle’ and ‘Membrane’ were highly enriched.

The over-represented (*P* value < 0.05) GO terms, including ‘Cell wall metabolism’ process, were enriched in both sets of DEGs from the two tissues of each soybean. However, some striking differences were observed between the over-represented GO terms of roots and leaves **[see Supporting Information—Table S2]**. For example, GO terms related to hydrogen peroxide catabolic process and cellular oxidant detoxification processes were preferentially enriched in the roots. By contrast, GO terms related to the regulation of jasmonic acid (JA)-mediated signalling pathway and cellular cation homeostasis were highly enriched in the leaves.

**Figure 4.**
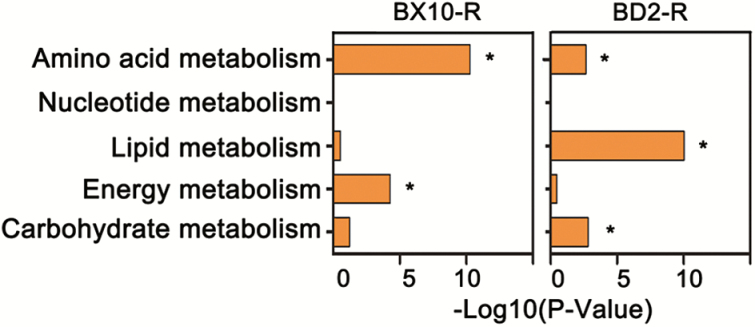
KEGG enriched pathways of DEGs in the roots of BX10 and BD2. Asterisk (*) indicates significant difference at *P* < 0.05.

Kyoto Encyclopedia of Genes and Genomes pathway enrichment analysis revealed that genes involved in ‘Amino acid metabolism’ pathway were enriched in the roots of both soybean genotypes (Fig. 4). By contrast, the genes involved in ‘Carbohydrate metabolism’ and ‘Lipid metabolism’ pathways were prefereially enriched in the roots of BD2 (Fig. 4).

### Effects of Al stress on the expression of genes involved in organic acid metabolism and exudation in roots

The exudation of organic acid from roots is required for Al tolerance in soybeans (Nian *et al.* 2007; Xu *et al.* 2010). The genes, including citrate synthase gene (*CS*) and malate dehydrogenase gene (*MDH*), directly involved in citrate cycle were not responsive to 48 h Al stress in the roots of these two soybean genotypes. This finding suggests that organic acid biosynthesis is not a rate-limiting step for the exudation of organic acids in the roots of both soybean genotypes, which has been observed in some other plant species, such as triticale (Hayes and Ma 2003), buckwheat (Zhu *et al.* 2015) and maize roots (Maron *et al.* 2008). However, the genes that encode pyruvate kinase (PK) and phosphoenolpyruvate carboxylase (PEPC) had up-regulated expression levels. The expression of gene encoding ATP-citrate lyase (ACL) was down-regulated only in BX10. In addition, the expression of gene encoding mitochondrial glutamate-oxaloacetate transaminase 2 (GOT2) was induced in the roots of BX10. However, these four genes showed no difference in BD2 (Table 2). These findings revealed that outside the mitochondrion, different types of organic acid metabolism occurred between the roots of BX10 and BD2.

**Table 2.** Expression of genes associated with citrate metabolism and exudation in roots. ^a^PK, pyruvate kinase; PEPC, phosphoenolpyruvate carboxylase; ACL, ATP-citrate lyase; FRD3b, ferric reductase defective3b; MATE, multidrug and toxic compound extrusion; MMC, mitochondrialoxoglutarate/malatecarrierprotein; GOT2, aspartate aminotransferase. ^b^BX10R and BD2R denote the roots of BX10 and BD2, respectively. ^c^Fold change, means the ratio of Al treatment vs. control.

Gene ID	Annotation^a^	BX10R^b^	BX10R	Fold change^c^	BD2R	BD2R	Fold change
		−Al	+Al		−Al	+Al	
Citrate metabolism							
glyma10g34490	PK	372.26	815.17	2.19	121.83	123.83	1.02
glyma12g33820	PEPC	118.74	562.77	4.74	130.48	229.15	1.76
glyma08g17010	ACL	433.24	179.49	0.41	723.23	685.12	0.95
Citrate transport							
glyma09g15550	FRD3b	574.44	390.76	0.68	1996.88	734.89	0.37
glyma13g27300	MATE	217.15	13497.02	62.15	12.10	15846.95	1309.98
glyma02g31370	MATE	3336.45	12374.30	3.71	717.18	8632.32	12.04
glyma01g02950	MMC	502.77	955.39	1.90	475.24	1051.99	2.21
Amino acid metabolism							
glyma01g32360	GOT2	284.54	681.49	2.40	194.42	225.67	1.16

Multidrug and toxic compound extrusion family plays an important role in transporting organic acids during Al stress. A previous genome-wide analysis found 117 MATE transporter genes in soybean, some of which were responsive to Al stress (Liu *et al.* 2016). In the present study, we screened three MATE genes in the roots of two soybean genotypes (Table 2). Glyma09g15550 (*GmFRD3b*), an *AtFRD3* homolog, was significantly down-regulated only in BD2. However, glyma13g27300 and glyma02g31370 were sharply up-regulated in both soybean genotypes under 48 h Al stress, specifically to a larger extent in BD2.

### Effects of Al stress on the expression of genes involved in JA biosynthesis and signalling in leaves

Two genes (glyma08g20200 and glyma13g31280) encoding lipoxygenase (LOX) were only significantly induced in BD2 leaves (Table 3). Similarly, genes encoding 12-oxophytodienoate reductase (OPR) and acyl-CoA oxidase (ACX) were also significantly up-regulated only in BD2 leaves. However, these genes were not induced in BX10 leaves.

**Table 3.** Expression of genes associated with JA biosynthesis and signalling pathway. ^a^LOX, lipoxygenase; OPR, 12-oxophytodienoate reductase; ACX, acyl-CoA oxidase; JAZ, jasmonate ZIM-domain protein. ^b^BX10L and BD2L denote the leaves of BX10 and BD2, respectively. ^c^Fold change means the ratio of Al treatment vs. control.

Gene ID	Annotation^a^	BX10L^b^	BX10L	Fold change^c^	BD2L	BD2L	Fold change
		−Al	+Al		−Al	+Al	
JA biosynthesis							
glyma08g20200	LOX	54.30	49.50	0.91	105.84	213.23	2.01
glyma13g31280	LOX	44.12	29.17	0.66	101.77	207.33	2.04
glyma13g16950	OPR	3.39	3.54	1.04	68.19	222.07	3.26
glyma05g31390	ACX	463.84	855.65	1.84	467.12	947.23	2.03
JA signalling							
glyma15g09980	JAZ	3.39	18.56	5.47	29.51	166.06	5.63
glyma13g17180	JAZ	133.49	563.07	4.22	610.62	2266.88	3.71
glyma17g05540	JAZ	99.56	158.22	1.59	374.51	838.16	2.24
glyma11g04130	JAZ	50.91	175.02	3.44	317.52	1694.02	5.34
glyma01g41290	JAZ	71.27	214.80	3.01	582.12	2381.84	4.09
glyma09g08290	JAZ	150.46	540.97	3.60	643.19	2097.87	3.26
glyma15g19840	JAZ	238.71	416.33	1.74	884.38	2340.57	2.65

As the key repressors of JA signalling pathway, jasmonate ZIM-domain proteins (JAZs) belong to the TIFY transcription factor family (Vanholme *et al.* 2007). We found that seven TIFY genes showed differential expression in both soybean genotypes (Table 3). These seven TIFY genes were all induced in BD2, whereas only four of them were induced in BX10. Moreover, the basal expression levels of these genes in BD2 were significantly higher than that in BX10.

### Real-time PCR validation of RNA-seq data

Ten randomly selected genes displaying diverse expression profiles were analysed by real-time PCR to validate the expression pattern of the DEGs. Among these genes, three were unresponsive to Al stress (FDR > 1), whereas the other seven showed differential expression (FDR < 0.05) both in the roots of BX10 and BD2 according to the 2-fold ratio and FDR < 0.05 thresholds. We found similar patterns between mRNA-seq and real-time PCR. Two sets of data revealed a significant correlation (*R*^2^ = 0.94) **[see Supporting Information—Fig. S2]**, indicating the reliability of the high-throughput sequencing data.

## Discussion

### Global responses of genes in different tissues of two soybean genotypes

We investigated the expression profiling of genes in the roots and leaves of two soybean genotypes under Al treatments by using Illumina HiSeq 2000 sequencing platform. Comparative analysis revealed that more genes in the root tips showed responsiveness to Al than those in the leaves in both soybean genotypes. This analysis supports the observation that root is the primary target of Al toxicity (Kollmeier *et al.* 2000). Moreover, more genes were up-regulated in roots than in leaves, suggesting that the roots might be more active than the leaves in response to Al stress.

Kyoto Encyclopedia of Genes and Genomes analysis revealed that genes involved in amino acid metabolism were mostly up-regulated in the roots of these two soybean genotypes. The expression levels of the genes that encode specific amino acid biosynthesis were induced under various rhizotoxic ion (Al, Cu and Cd) stresses (Zhao *et al.* 2010). In addition, increasing amino acid accumulation is considered as a hallmark of Al-toxicity alleviation (Wang *et al.* 2015). Therefore, the activation of amino acid metabolism could be a common mechanism of Al tolerance in these two soybean genotypes.

Kyoto Encyclopedia of Genes and Genomes analysis also revealed that the expression levels of the genes implicated in lipid biosynthesis were up-regulated in the roots of BD2. Maintaining the activities of lipid biosynthesis-associated enzymes and the stability of lipid composition is required for Al resistance in rice (Huynh *et al.* 2012). One gene encodes GDSL-like lipase and functions as lipid hydrolysis; this gene was strikingly induced in BX10 and BD2 with a larger extent in BD2, which supported the observation that a large decrease in lipid content is correlated with Al sensitivity (Huynh *et al.* 2012). Therefore, the induction of lipid metabolism-related genes appears to be a passive measure for the turnover of lipid to maintain the stability of membrane lipid composition.

### Differential citrate metabolism in the roots of two soybean genotypes

Al-induced citrate and malate exudation are critical for Al resistance in soybeans (Yang and Zheng 2006; Liang *et al.* 2013). Thus, the induction of genes encoding citrate transporters is required for Al-stimulated citrate efflux (Yang and Zheng 2006). Citrate can be secreted into rhizosphere and/or vascular system for Al chelation. The function of MATE proteins as mediators of citrate transport is conserved in different plant species (Magalhaes *et al.* 2007; Zhou *et al.* 2013). Glyma13g27300, a MATE gene that is considered as a candidate gene involved in Al tolerance in soybeans (Liu *et al.* 2016), was significantly up-regulated in our study. In addition, the down-regulation of another MATE gene, *GmFRD3b*, received our attention. Previous report revealed that both *GmFRD3a* and *GmFRD3b* are involved in Fe deficiency response in soybeans (Rogers *et al.* 2009; Takanashi *et al.* 2014). Further study showed that *GmFRD3a* is up-regulated in response to Al (You *et al.* 2011). In the present study, we found that *GmFRD3a* was not induced in these two soybean genotypes, instead, *GmFRD3b* was down-regulated in the roots of BD2 but not in BX10. In *Arabidopsis*, AtFRD3 functions in transporting citrate into root vasculature where it forms a complex with iron and then transfers to shoots (Rogers and Guerinot 2002; Green and Rogers 2004; Durrett *et al.* 2007). Moreover, the overexpression of *AtFRD3* contributes to Al tolerance (Durrett *et al.* 2007). Therefore, the up-regulation of glyma13g27300 and glyma02g31370 in both genotypes and the down-regulation of *GmFRD3b* in BD2 roots suggest that the enhanced citrate exudation is a common mechanism, whereas the sensitive soybeans increase the supply of citrate for exudation by inhibiting the internal flow of citrate into xylem. We suggest that the decreased expression of *GmFRD3b* in BD2 may suppress citrate loading into root xylem. As a result, a relatively large amount of toxic Al would be translocated into the aerial portions, accumulate and cause Al toxicity in the leaves.

**Figure 5.**
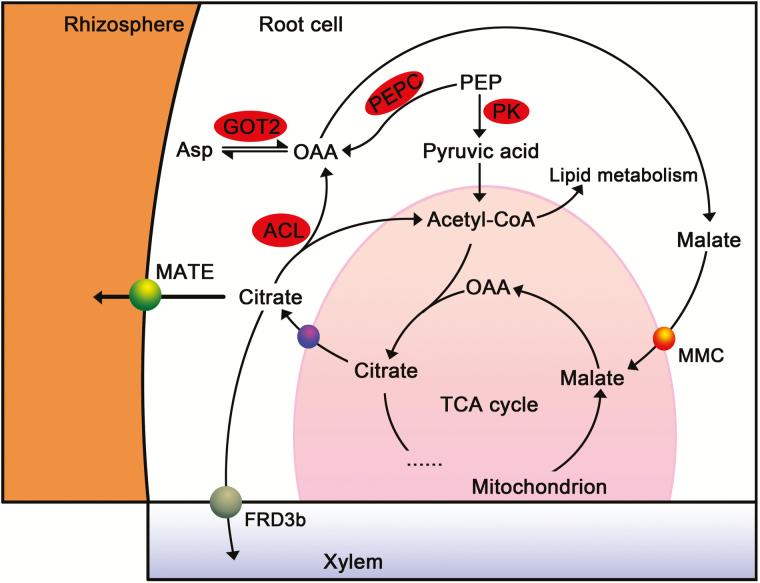
Schematic network reveals the differences in organic acid metabolism in the roots of two soybean genotypes. Genes differentially expressed in BX10 are highlighted in red oval. Transporters are shown in coloured spheres.

ALS3 is localized to the plasma membrane of phloem, which transports Al into phloem and then moving it away from the sensitive tissues to maintain root growth in *Arabidopsis* (Larsen *et al.* 2005). We noticed that two *ALS3* genes, glyma03g33290 and glyma10g05420, were highly up-regulated by Al stress in the roots of both soybean genotypes, specifically to a relatively large extent in BD2 **[see Supporting Information—Fig. S3]**. This finding suggests that the up-regulation of *ALS3* is a possible mechanism in response to Al stress in different soybean genotypes. However, the long-distance transfer of Al through the phloem transport system would be more promoted in BD2 than in BX10. Phloem transport consumes energy (Dong and Zhang 1986), which suggests that more ATP may be used by BD2 to redistribute Al away from the root tip. The altered organic acid metabolism in some plant species has been addressed in response to Al stress (de Carvalho Gonçalves *et al.* 2005; Yang *et al.* 2011). In BX10, genes encoding PK and PEPC were up-regulated, which suggested that the transformation from phosphoenolpyruvate (PEP) to pyruvic acid and PEP to oxaloacetic acid (OAA) was enhanced (Fig. 5). Increased pyruvic acid and OAA then promote the flow of tricarboxylic acid (TCA) cycle. Additionally, we found that the expression of *ACL* gene was down-regulated in BX10 (Fig. 5). ACL catalyses the cleavage of citrate into two products: OAA and acetyl-CoA. During this process, OAA can be used for the anaplerotic reaction of TCA cycle while acetyl-CoA is required for lipogenesis (Takeda 1969). Therefore, the decrease in *ACL* gene expression implicated that the degradation of citrate in cytosol will be suppressed in BX10. By contrast, the dissolution of extramitochondrial citrate was not inhibited in BD2, which suggested that the amount of citrate available for secreting was less in BD2 than that in BX10. Genes involved in lipid metabolism were over-represented in the roots of BD2, which suggested that a large amount of acetyle-CoA would be recruited to facilitate fatty acid biosynthesis. Thus, few acetyle-CoA can be utilized for citrate biosynthesis. This condition can retard the TCA cycle in BD2 roots. In addition, the expression of *GOT2*, which catalyses the reversible conversion between aspartate and OAA, was up-regulated in BX10 roots. This finding indicated that organic acid metabolism could be favoured by the extra supply of OAA produced from the enhanced amino acid metabolism. Therefore, we deduced that metabolism might be one of the leading causes of Al sensitivity in BD2.

### Differential JA biosynthesis and signalling in the leaves of two soybean genotypes

Jasmonic acid is a phytohormone that functions in the signalling of defence response (Howe 2001). JA can be induced by various stimuli (Gao *et al.* 2004; Glazebrook 2005; Maksymiec *et al.* 2006; Hess 2010). JA-mediated regulation of defence-associated genes in leaves is an essential part of self-defence mechanism in response to adverse circumstances (Omer *et al.* 2000; Henkes *et al.* 2008; Black *et al.* 2009). The proteins, including LOX, OPR and ACX, in *Citrus* species were up-regulated in roots under Al stress (Jiang *et al.* 2015). A latest study reported that JA can enhance the Al-induced root growth inhibition in *Arabidopsis* (Yang *et al.* 2017). These findings revealed that in roots, JA signalling was involved in Al stress. However, we observed that the genes, including *LOX*, *OPR* and *ACX*, involved in JA biosynthesis were significantly induced in the leaves of BD2. Increased JA expression can serve as an important signalling molecule to initiate defence response by activating the gene expression levels (Blée 2002). These genes involved in JA biosynthesis were not induced in the leaves of BX10, which suggested that the intensity of Al stress was insufficient to trigger the JA response in BX10.

JAZs are involved in JA signalling transduction pathway as transcription repressors (Pauwels and Goossens 2011). Under stress condition, increased bioactive JA (JA-Ile conjugate) guides the binding of JAZs to SCF^COI1^ ubiquitin E3 ligase complex to initiate the 26S proteasome degradation process. After the destruction of JAZs, transcription factors are released from JAZ-mediated repression, and the subsequent activation of JA-responsive genes is allowed (Pauwels and Goossens 2011). We found that seven *JAZ* genes were induced in the leaves of BD2, whereas three of them were induced in the leaves of BX10. The dissolution and induction of *JAZ* genes have at least two functions: facilitating the activation of defence genes and attenuating the following JA response, which may be essential for maintaining cellular stability because the long-term stimulation of defence response may result in damage (Thines 2007). In BD2 leaves, the up-regulation of genes involved in JA biosynthesis and the resulting induction of *JAZ*s suggested that JA defence response was activated. Given that long-term stress response requires resource consumption, and the decrease in ATP biosynthesis may impair Al tolerance of plants (Honda *et al.* 1997; Hamilton *et al.* 2001), the activated expression of JA-associated genes would inevitably cause growth arrest in leaf, thereby weakening its adaptation to Al toxicity in BD2.

### Synergistic effect between roots and leaves in response to Al stress

**Figure 6.**
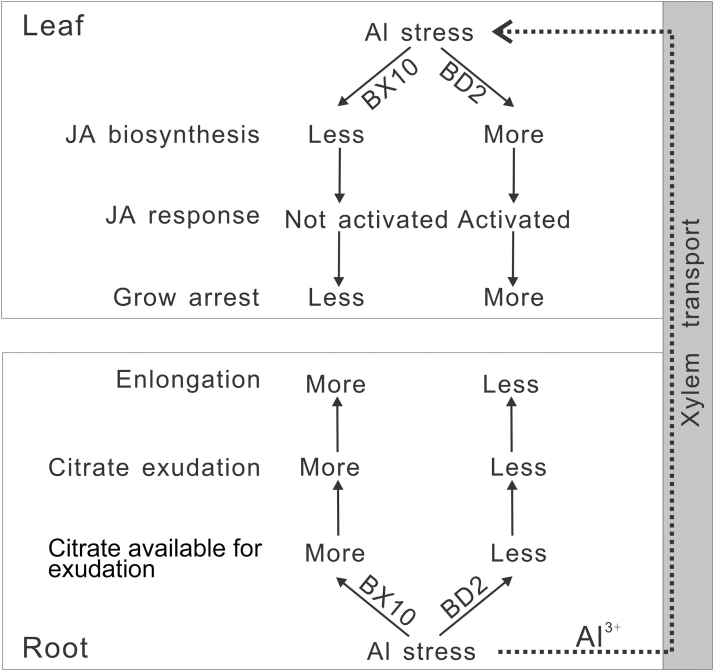
Proposed model for Al-induced citrate metabolism and secretion in the roots, and JA biosynthesis and signalling in the leaves of BX10 and BD2. In BX10, genes involved in citrate metabolism and exudation were induced in the roots. This finding suggested that more citrate could be secreted into rhizosphere for Al chelation, which is very essential for alleviating the Al toxicity in roots. Toxic Al^3+^ was continuously translocated through apoplastic and/or symplastic pathways. Genes involved in JA biosynthesis and signalling were highly induced in the leaves because much more Al^3+^ were accumulated in the leaves of sensitive soybean genotype. This finding indicated that JA-mediated defence response was activated, which could lead to resource and energy expenditure and growth arrest of leaves. These conditions are signs of Al toxicity.

Growth correlation between the below-ground and above-ground structures of plants is a common physiological phenomenon. We deduced that a unique cooperation scenario occurred between the roots and leaves of the two soybeans in response to Al stress (Fig. 6). Citrate biosynthesis might not be activated in the roots of BD2, which is supported by the previous findings that citrate exudation is lower in BD2 than in BX10 (Dong *et al.* 2004). Although the citrate transport activity was higher in BD2 roots, this phenomenon is considered as a compensation measure to resolve the shortage of citrate biosynthesis. The internal transport of citrate into xylem might be inhibited in the roots of BD2. Therefore, Al could not be sufficiently chelated by citrate in its root vasculature. As a consequence, a large amount of toxic Al would escape and finally accumulate in the leaves of BD2. Excessive Al may lead to JA biosynthesis via the induction of related genes. High accumulation of JA activates JA response, which results in the growth inhibition of leaves (Ueda *et al.* 1995; Ulloa *et al.* 2002; Liu *et al.* 2010), because JA defence response allows resource diversion (Zavala and Baldwin 2006) and leads to ATP biosynthesis deficiency (Ruiz-May *et al.* 2011).

## Conclusions

We investigated the molecular mechanisms of different Al tolerance in two contrasting soybean genotypes through the global transcriptome analysis of the roots and leaves. Our RNA-seq data reveal that the genes involved in citrate metabolism and secretion are preferentially expressed in the roots of BX10. The genes implicated in JA biosynthesis and signalling are highly induced in the leaves of BD2. These findings suggest that on one hand, BX10 can secrete additional citrate into rhizosphere from the roots to chelate Al. On the other hand, BX10 can avoid JA-mediated defence response that allows resource allocation to maintain leaf growth. Our results provide new insights into the understanding of the molecular mechanisms of Al tolerance in different tissues of soybeans.

## Supporting Information

The following additional information is available in the online version of this article—

**Table S1.** Differentially expressed genes in each category.

**Table S2.** Over-represented GO terms in roots and leaves.

**Figure S1.** Time course of root length of BX10 and BD2 soybeans under 0 or 50 μM Al^3+^ treatments. Error bars denote mean ± SD (*n* = 15).

**Figure S2.** Correlation analysis of real-time PCR data and mRNA-seq data. The ratio of each gene expression (log2) in the mRNA-seq data was calculated and plotted against the ratio calculated in the real-time PCR data.

**Figure S3.** The expression patterns of *ALS3* genes in the roots of two soybean genotypes.

Supporting-Information

## Sources of Funding

This research was supported by the National Key Research and Development Program of China (2016YFD0101005), the grants from the National Natural Science Foundation of China (NSFC) (31271744, 31372140), the Program for Changjiang Scholars and Innovative Research Team in University (IRT_14R27) and the Important National Science & Technology Specific Project (2016ZX08011-003, 2014ZX08011-003).

## Contributions by the Authors

C.-Y.T., J.-L.Q. and Y.-H.Y. designed research; S.-C.H., S.-J.C., Y.-M.G., Y.-J.J., D.-Q.H. and J.C. performed research; S.-C.H., G.-H.L., R.-W.Y., C.-Y.T., J.-L.Q. and Y.-H.Y. analysed data; S.-C.H., C.-Y.T., J.-L.Q. and Y.-H.Y. wrote the paper. All authors have read and approved the final version of the manuscript.

## Conflicts of Interest

None declared.
